# Editorial: Psychosexual health and sexuality: multi-disciplinary considerations in clinical practice

**DOI:** 10.3389/fpsyt.2023.1244139

**Published:** 2023-07-26

**Authors:** Henrique Pereira

**Affiliations:** ^1^Department of Psychology and Education, Faculty of Social and Human Sciences, University of Beira Interior, Covilhã, Portugal; ^2^Research Centre in Sports Sciences, Health Sciences and Human Development (CIDESD), Vila Real, Portugal

**Keywords:** psychosexual health, sex therapy, human sexuality, sexual minorities, COVID-19 pandemic on sexuality and sexual health

Human sexuality describes a global experience, framed in a given historical and sociocultural context of human evolution, which involves intimacy, social contact, procreation, sexual function, and sexual problems. The World Health Organization defines it as an integral human right, including sexual and reproductive rights and their respectful expression, not restricted to the absence of sexual problems. In this sense, the importance of considering the construct of psychosexual health is growing, integrating physical, psychological, and wellbeing aspects in the expression of people's sexuality, as well as openness to formal and systematized research sources, which will help to consolidate different contributions with applications to clinical practice. It is based on this premise that the creation of this thematic issue arose.

Resistance seems to persist when focusing on the study of human sexuality from a multidisciplinary perspective, with integration efforts of the different scientific contributions. This probably has to do with the need to optimize resources, but the truth is that there are countless intersections between psychological health and wellbeing and human sexual expression. An integrated view of human sexuality between psychology, biology, medicine, and sociology will help make this approach more visible and effective.

This Research Topic thus constitutes a tool to create a platform for sharing various contributions in a multidisciplinary way. Nine original articles, six quantitative articles, two systematic literature reviews, and a case report were received.

One such study (Buchholz et al.) aimed to assess the degree of correlation of prenatal androgen markers with online sexual compulsiveness and erectile function in young men, recruiting 4,370 participants. The results indicated that lower androgen levels were associated with higher levels of online sex compulsiveness, as well as a higher spermarche age correlated with higher levels of online sex compulsiveness. On the other hand, the more sexual compulsion, the lower the erectile function and ejaculatory control. These results allowed us to demonstrate the importance of intrauterine predisposition in mediating sexual behavior in adult men.

Another study (Hatta et al.) evaluated the psychosocial determinants of marital satisfaction among gynecological cancer survivors in 116 Malaysian patients. If, on the one hand, 37.9% of the participants said they had low levels of marital satisfaction, whereas psychosocial determinants (such as low education) were found to be associated with low marital satisfaction. Thus, this study demonstrated that sexual dysfunction and low levels of education can interfere with marital satisfaction among gynecological cancer survivors.

The third study included in this Research Topic assessed levels of quality of life and sexual health in an Indian city during the COVID-19 pandemic (Chatterjee et al.). In total, 1,376 individuals participated, of which 27.18% had sexual problems. From the point of view of emotional functioning, moderate levels of depression and anxiety were found, and older people and women showed greater worsening of sexual problems. This study contributed to demonstrate the importance of mental health in sexual functioning and quality of life.

The fourth contribution is the presentation of a case report whose purpose was to assess the challenges of transgender people in China (Shi et al.). The authors reinforce the idea that great progress has been seen in recent times and that they describe an important advancement in the protection of the rights of trans people, citing the example of people who go to the local police to request a new identity card after undergoing gender reassignment surgery. This mechanism represents a way of protecting the rights of trans people in China, allowing the transition to experience dignified gender expression, which also increases the visibility of the trans community and helps in the fight against transphobia in the country.

Another study (Cai et al.) evaluated the mental health of children with sexual development problems, comparing 30 children with problems and 30 children without problems, measuring their levels of anxiety, depression, and childhood memories. The results showed that children with problems had more anxiety and more depression, which are probably linked to variables related to family dynamics. The authors draw attention to the importance of creating an emotionally stable environment for children with developmental sexual problems to regulate emotional problems.

The following study (Zheng et al.) evaluated 1,142 patients with erectile dysfunction from a hospital in China, with the objective of determining the predictors of the patient's health. Erectile function, physical pain, and frequent and prolonged urination were the assessed determinants, and the authors confirmed a nomogram to predict the low and high risk of disease in patients with erectile dysfunction.

The seventh study (Cervilla and Sierra) compared the differences between men and women regarding masturbation parameters and their relationship with satisfaction with orgasm. The study demonstrated several differences between men and women, including men who masturbated earlier for the first time, masturbated more frequently, and expressed greater solitary sexual desire. Women, on the other hand, demonstrated greater intensity in the subjective experience of orgasm. However, for both men and women, the affective dimension proved to be decisive in the use of masturbation, highlighting its importance in sexual functioning, even in a relational context.

A systematic review and meta-analyses on the effectiveness of school-based child sexual abuse intervention among school children in the new millennium era was the topic of the eighth contribution (Che Yusof et al.). In total, 29 studies assessing knowledge, skills, and attitudes were analyzed, and the authors concluded that programs that work on these domains are effective, which translates into improved wellbeing of children victims of sexual abuse.

Finally, the last study (Mourikis et al.) was also a systematic review of adult sexual wellbeing and behavior during the COVID-19 pandemic. A total of 31,911 participants were assessed for changes in sexual functioning attributable to the COVID-19 pandemic. Data analysis models demonstrated the negative impact of the pandemic on female sexual function but not on male subjects.

We believe that the contributions compiled in this Research Topic are important reinforcements for the advancement of scientific knowledge in the field of psychosexual health, with implications for clinical practice. On the one hand, psychosexual health is an integral part of psychological wellbeing, and on the other hand, it is necessary that all technicians and professionals working in this area understand the challenges and concerns and accept these contributions as a form of continuous improvement of their integrative practices, improving their effectiveness. In fact, many professionals will be able to benefit from this integrative vision as it will allow a paradigm shift, focusing on the multidisciplinary aspects of human sexuality.

## Author contributions

The author confirms being the sole contributor of this work and has approved it for publication.

